# Numerical and experimental analysis of Lagrangian dispersion in two-dimensional chaotic flows

**DOI:** 10.1038/s41598-022-11350-1

**Published:** 2022-05-06

**Authors:** Giovanni La Forgia, Davide Cavaliere, Stefania Espa, Federico Falcini, Guglielmo Lacorata

**Affiliations:** 1 University of Cassino and Southern Lazio, 03043 Cassino, FR Italy; 2 CNR, Institute of Marine Sciences, Via Fosso del Cavaliere 100, 00133 Rome, Italy; 3 DICEA, University of Rome ’La Sapienza’, Via Eudossiana 18, 00184 Rome, Italy; 4 CETEMPS, Via Vetoio, 67100 L’Aquila, Italy

**Keywords:** Physics, Fluid dynamics, Statistical physics, thermodynamics and nonlinear dynamics

## Abstract

We present a review and a new assessment of the Lagrangian dispersion properties of a 2D model of chaotic advection and diffusion in a regular lattice of non stationary kinematic eddies. This model represents an ideal case for which it is possible to analyze the same system from three different perspectives: theory, modelling and experiments. At this regard, we examine absolute and relative Lagrangian dispersion for a kinematic flow, a hydrodynamic model (Delft3D), and a laboratory experiment, in terms of established dynamical system techniques, such as the measure of (Lagrangian) finite-scale Lyapunov exponents (FSLE). The new main results concern: (i) an experimental verification of the scale-dependent dispersion properties of the chaotic advection and diffusion model here considered; (ii) a qualitative and quantitative assessment of the hydro-dynamical Lagrangian simulations. The latter, even though obtained for an idealized open flow configuration, contributes to the overall validation of the computational features of the Delft3D model.

## Introduction

Nonlinear dynamical features, like chaos or turbulence, are known to play a major role as physical mechanisms of transport and dispersion of Lagrangian trajectories in fluids like, e.g., the ocean or the atmosphere. Chaotic advection, or Lagrangian chaos, is quite common in natural dynamical systems since it is present even in regular, i.e. non turbulent, velocity fields^1–4^.

Numerical simulations of a Lagrangian tracer drifting away on the ocean surface, for instance, are normally affected by a finite predictability time, due to the forecast error sensitivity to both initial conditions and uncertainties in the model dynamics^5–7^ (e.g., finite resolution, parameter values, etc.). When dealing with Lagrangian trajectory models, in general, two major issues arise: (i) assessing the accuracy of the numerical trajectories; (ii) studying possible solutions to optimize the model. These two aspects are the foundations of a recently introduced Lagrangian validation methodology, based on a dynamical systems approach^7,8^.

Hydro-dynamical models are usually subject to Eulerian-type validations consisting, substantially, in the comparison of a vector or scalar field computed by the model with the corresponding field obtained from experimental or observational data^9^. Evaluating the accuracy of Lagrangian trajectory simulations requires an independent direct approach: even small differences, indeed, between two non linear velocity fields, e.g. in the small-scale energy spectrum or in the time variability frequency, may lead to completely different trajectories evolution^3,7,8^.

In this context, it is worth stressing that, unlike the case of large-scale models of ocean surface circulation, for which numerical trajectories can be compared directly to satellite-tracked real drifter data^8,10,11^, the issue affecting hydrodynamic models, normally used for small-scale coastal applications and bounded flow configurations, is the paucity of experimental or observational Lagrangian data, essential to test the consistency of numerical trajectories. In spite of the growing interest in studies concerning, e.g., the dispersion of pollutants, nutrients or sediments in coastal areas and river-sea interface systems^12–15^, the validation of hydro-dynamical Lagrangian simulations still remains an open question.

The kinematic model, here representing the case study, is a variation of the well known Rayleigh–Bénard convection model, introduced by Solomon and Gollub^16^. The model stream-function defines a time-dependent, spatially periodic, 2D regular lattice of square cells. Here the stream-function plays the role of Hamiltonian function: in stationary conditions, two-dimensional trajectory motion is necessarily regular and evolves along closed stable orbits inside the cells; for weak time-dependent perturbations, a thin chaotic layer forms around the separatrices while the innermost orbits are kept regular by the KAM tori; for a suitable choice of the perturbative parameters, all orbits become open and unstable and chaos propagates to all available space^3,17^.

In the latter case, trajectory pair dispersion is characterized by two major regimes: exponential separation (below the cell size) and standard diffusion (above the cell size). This scenario appears quite often when analyzing real ocean Lagrangian drifter dispersion (possibly enriched by the presence of a third, mesoscale, super-diffusive regime due to turbulence)^11^.

Recently, there has been a renewed interest in the use of chaotic advection and diffusion systems as models of Lagrangian turbulence^18^, which are proved to be very effective, e.g., as kinematic models of “sub-grid”, unresolved velocity modes in large-scale numerical trajectory simulations computed from ocean circulation fields^7,8^.

While this class of models have been widely reviewed and analyzed in numerous theoretical and computational studies, laboratory experiments remain, nevertheless, essential to establish physical relevance, validity, and reproducibility of predicted phenomena^4,19–21^. At this regard, we present here a full analysis of absolute and relative dispersion properties of our chaotic advection and diffusion case study, examined from three different perspectives: kinematic flow, hydrodynamic model, and laboratory tank experiment.

## Results

Large datasets of trajectory pairs (${\sim }10^4$) were evaluated for each of the three systems under consideration, i.e., kinematic convection model, hydrodynamic model and laboratory tank experiment, hereinafter referred to as Kin2D, Delft3D and LabExp, respectively.

For all systems, one-particle and two-particle dispersion statistics were analyzed in terms of dynamical systems techniques: variance of particle displacement with respect to the release position and Lagrangian velocity auto-correlation functions, as regards the absolute dispersion, and finite-scale Lyapunov exponents (FSLE), as measure of the scale-dependent relative dispersion rates (see “Methods” section for more details).

From the data analysis, some important physical characteristics like, e.g., auto-correlation time scale, spatial correlation length, Lagrangian maximum Lyapunov exponent, diffusion coefficient, were evaluated and discussed to establish a qualitative and quantitative comparison between models and experiments. At this regard, it is necessary to give a kind of “operating definition” of these physical quantities, valid for all systems analyzed in the present work.

We agree to consider the Lagrangian auto-correlation time scale, $\tau_{C}$, as the order of magnitude of the time interval after which the auto-correlation function lies definitely below a (conventionally chosen) threshold of $\pm 20\%$ of the initial value. This time scale corresponds, approximately, to the knee of the second-order absolute dispersion moment, at the transition between ballistic and diffusive regime. The spatial correlation scale, $L_C$, is defined as the pair separation scale (as order of magnitude) corresponding to the knee of the FSLE curve, at the transition between chaos and diffusion in the relative dispersion process. The Lagrangian maximum Lyapunov exponent, $\lambda_{L}$, is estimated as the plateau level of the FSLE at small separation scales^22^. The effective (eddy) diffusion coefficient, $D_E$, is given, as order of magnitude, by the value of the coefficient of the $\delta ^{-2}$ law that best fits the FSLE data at large separation scales^23^.

Here below, we report a brief introduction of the three systems and the results obtained for each of them.

### Kinematic eddy model

As previously mentioned, our case study consists of a 2D regular lattice of non stationary kinematic eddies (Kin2D, hereafter). This type of model has been recently developed and applied, in a multi-scale version, to simulate homogeneous and isotropic Lagrangian turbulence^18^ and as a sub-grid model of unresolved turbulent motions in Lagrangian simulations based on large-scale ocean current fields^7,8,24^.

The Kin2D velocity field components, *u* and *v*, are derived from a stream-function $\Psi$ according to the following equations:

1 $$\begin{aligned} \begin{aligned} \frac{dx_1}{dt}&= u(x_1,x_2,t) = \frac{\partial \Psi }{\partial x_2} \\ \frac{dx_2}{dt}&= v(x_1,x_2,t) = -\frac{\partial \Psi }{\partial x_1} \end{aligned} \end{aligned}$$

where $x_1$ and $x_2$ are the spatial coordinates of a fluid particle, *t* is the time, and

2 $$\begin{aligned} \Psi =\Psi (x_1,x_2,t)=\frac{\alpha }{k} sin\{k \cdot [x_1-\varepsilon \cdot sin(\omega \cdot t)]\} sin\{k \cdot [x_2-\varepsilon \cdot sin(\omega \cdot t + \phi )]\} , \end{aligned}$$

where $\alpha$ is the maximum velocity magnitude and $k = 2 \pi / L$ is the wave-number associated to the wavelength *L*. The Hamiltonian structure of (1) ensures that the kinematic model is a 2D conservative dynamical system. The presence of time-dependent terms in the stream-function (2) is a necessary condition for chaotic advection^1–3^.

**Figure 1:**
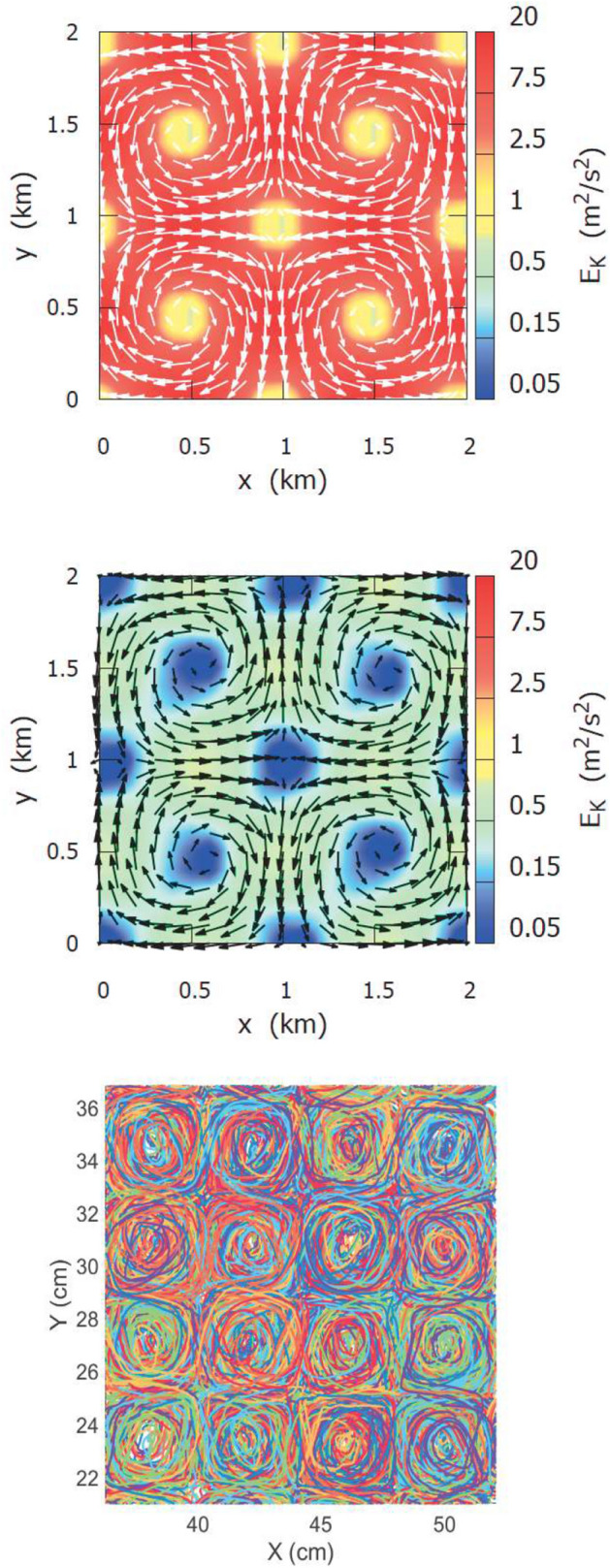
Mean kinematic velocity field, assimilated as input wind forcing with 10 m/s peak value (upper pannel) and mean hydrodynamic velocity field with $\sim$ 1 m/s peak value (center panel) of the Delft3D simulation. Bottom panel: Lagrangian trajectories obtained from laboratory experiments.

The kinematic velocity pattern is shaped like a 2D lattice of non-steady (i.e. subject to time periodic oscillations) square cells, of size *L*/2, with alternate vorticity, Fig. 1 (upper panel). The model set up is such that the eddy size *L*/2 is equal to 1 km and the maximum speed $\alpha$ is equal to 1 m/s. Last, the time oscillation parameters of the stream function, $\varepsilon$ and $\omega$, are suitably chosen in order to have a full chaotic regime of the Lagrangian flow, see Table 1 for more details. In the numerical simulations, the integration time step of the kinematic trajectories is $dt_{kin}=10$ s, about $10^3$ smaller than the expected Lagrangian characteristic time scale.

**Table 1:** Expected dispersion properties for the case study under consideration.

	Absolute dispersion	Relative dispersion
**Time range**		
$t \ll \tau _C$	$A_2(t) \sim \sigma ^2 t^2$	$R_2(t) \sim$ e$^{2 \lambda _L t}$
$t \gg \tau _C$	$A_2(t) \sim 2 D_E t$	$R_2(t) \sim 4 D_E t$
**Scale range**		
$\delta \ll L_C$	–	$\lambda (\delta ) \sim \lambda _L$
$\delta \gg L_C$	–	$\lambda (\delta ) \sim D_E\delta ^{-2}$

Notice: $\tau _C$ is the correlation time, $L_C$ is the correlation length, $\sigma ^2$ is the velocity variance, $D_E$ is the eddy-diffusion coefficient and $\lambda _L$ is the maximum Lagrangian Lyapunov exponent, $A_2(t)$ and $R_2(t)$ are the second order moments of absolute and relative dispersion, respectively, and $\lambda (\delta )$ is the FSLE. Relation to model parameters: $\tau _C \sim 2 \pi k^{-1}/\alpha$, $L_C \sim 2 \pi k^{-1}$, $\sigma ^2 \sim \alpha ^2$, $D_E \sim 2 \pi k^{-1} \alpha$, $\lambda _L \sim k \alpha / 2\pi$. The oscillation parameters are such that: $\varepsilon / (2 \pi k^{-1}) \sim 10\%$ and $\omega \sim \alpha / (2 \pi k^{-1})$. For the current set-up, the trajectory flow evolves in a full chaotic regime, after the complete destruction of the KAM tori.

**Figure 2:**
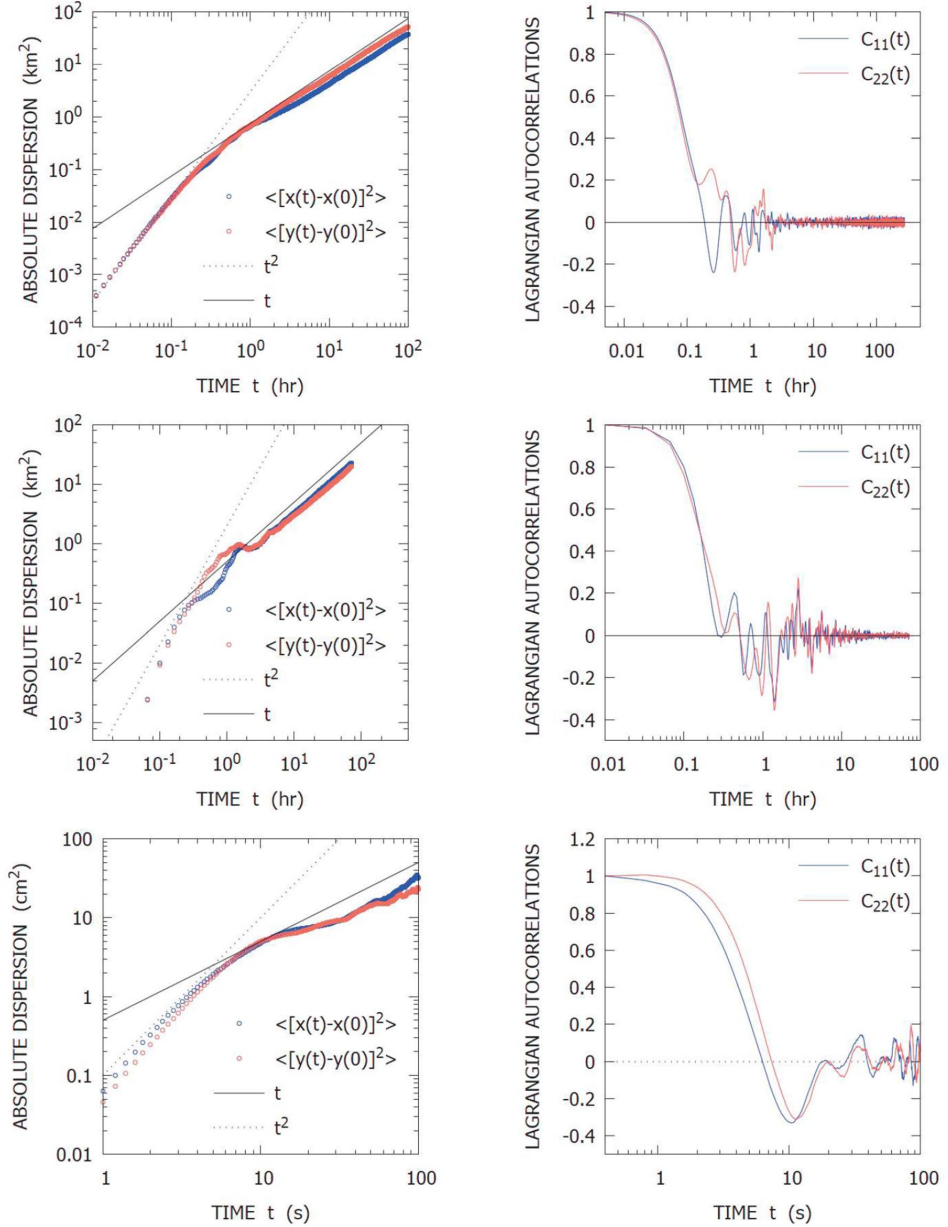
Single trajectory statistics: absolute dispersion and Lagrangian velocity autocorrelation for Kin2D (upper panel), Delft3D (center panel) and LabExp (lower panel).

**Figure 3:**
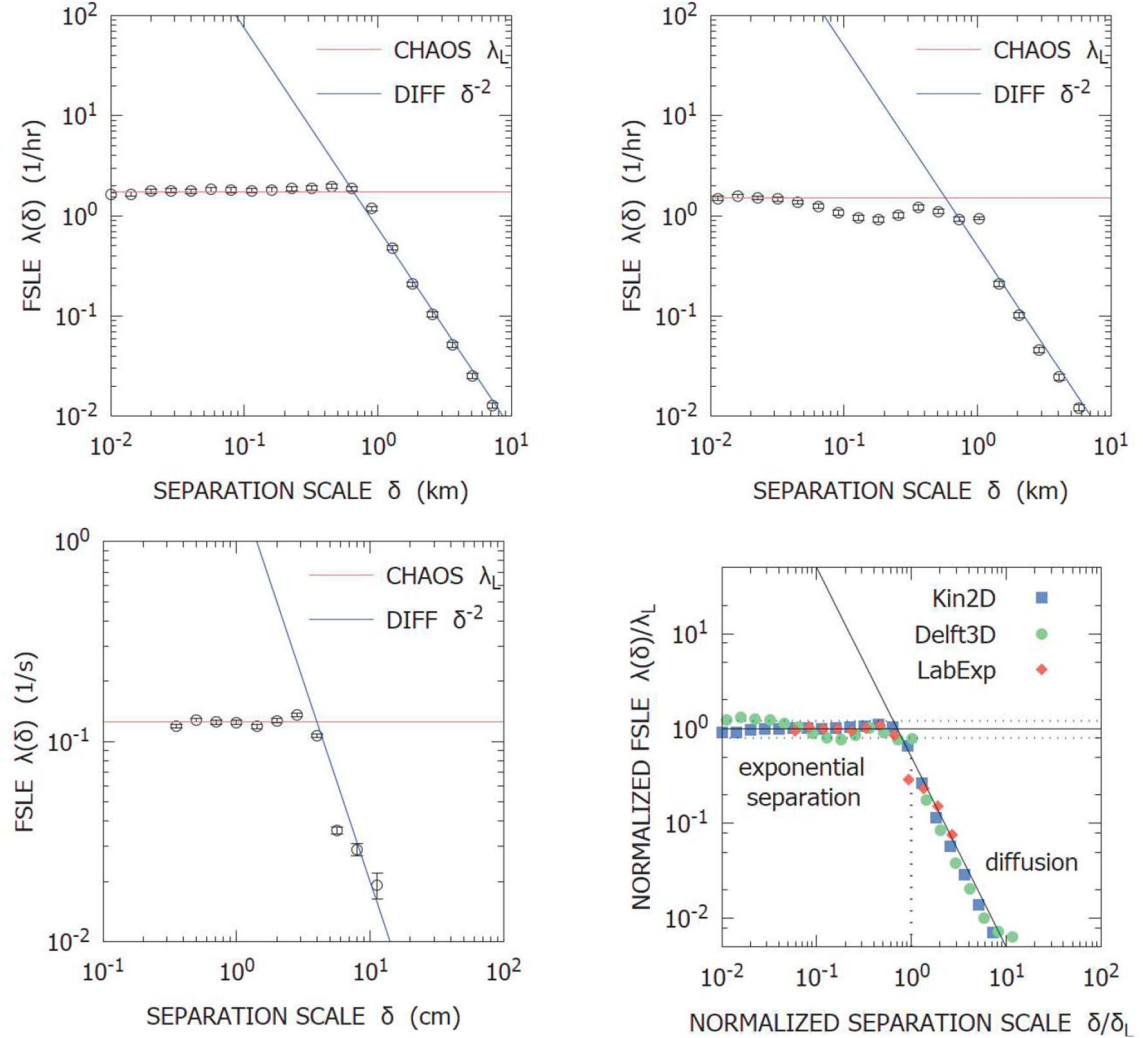
Finite-Scale Lyapunov Exponents: Kin2D (top left), Delft3D (top right), LabExp (bottom left) and overlap of all the renormalized functions (bottom right).

From a single-particle point of view, the system dynamics is statistically equivalent to a finite-time auto-correlated Brownian motion, i.e., with a mean ballistic-like homogeneous transport for times shorter than the auto-correlation time, and a standard diffusion for times much longer than the auto-correlation time, Fig. 2 (upper panels). Relative dispersion displays two regimes, Fig. 3 (top left panel), in accordance to the theoretical expectation, Table 1: (i) exponential separation, corresponding to Lagrangian chaos, for scales below the cell size; (ii) asymptotic standard diffusion for scales above the cell size.

At the end of the analysis, we observe that: (i) the Lagrangian correlation length is of the order of the cell size, $L_C \sim$1 km; (ii) the auto-correlation time scale is of the order of the eddy turnover time, $\tau _C \sim$ 1 h; (iii) the maximum Lagrangian Lyapunov exponent is of the order of the inverse turnover time, $\lambda _L \sim$ 1 h$^{-1}$, and (iv) the asymptotic eddy-diffusion coefficient is of the order of the square cell size divided by the turnover time, $D_E \sim L_C^2/\tau _C$.

Notice this correspondence between Lagrangian dispersion characteristics and flow parameters will be recurrent in all cases under examination.

### Delft3D hydro-dynamical model

The Delft3D^9,25^ model is a well known computational tool for modelling hydrodynamics in cases where the horizontal length and time scales are significantly larger than the vertical scales, e.g., shallow seas, coastal areas, estuaries, lagoons, rivers, and lakes^12,26,27^. For its characteristics, the Delft3D model is becoming a widespread modelling system in coastal oceanography and morpho-dynamics applications, thanks to its numerical stability and ease of use, compared to other platforms^28–30^.

In order to simulate a hydro-dynamical version of the kinematic model flow, the Kin2D velocity field is “strategically” assimilated as wind forcing in the Delft3D simulations. We decided to have a maximum hydro-dynamical speed very close to the maximum kinematic velocity, $\alpha$ (1 m/s). This implies that the wind peak value must be set to $10 \alpha$, because of the damping factor in the response of the hydrodynamic field to the forcing. The output hydro-dynamical current field displays a spatially periodic pattern of (non stationary) square convective cells, of size 1 km $\times$ 1 km, updated every $\Delta t_{out} = 6$ s on a grid with spatial resolution equal to $\Delta l_{grid} = 100$ m, Fig. 1 (upper and center panels), of shape very similar to that of the kinematic field. At this regard, we would like to stress that the parameter set up of the numerical models is, to some extent, arbitrary, and it is not meant to represent properly a realistic system.

Hence, $10^4$ numerical trajectory pairs were released with uniform distribution inside one cell in the middle of the domain. The initial pair separation was set to a small fraction, $10^{-2}$, of the cell size, and the duration of the simulation was 20 days, enough to span a distance comparable with the domain size ($\sim 20$ km). The integration time step of the particle tracking was $dt_{hydro}=12$ s, but for the analysis the time resolution of the trajectories was downgraded to 120 s.

Qualitatively, one-particle statistics is consistent with the scenario of time-correlated deterministic “Brownian-like” diffusion, Fig. 2 (center panels), i.e. oscillating auto-correlation functions with exponentially decaying envelope and transition, from ballistic regime to diffusive regime, of the second-order absolute dispersion moment in correspondence of the auto-correlation time scale; as regards two-particle statistics, the FSLE displays, as well, a transition between a nearly flat plateau at small separation scales and a typical diffusive scaling for large separation scales, in correspondence of the cell size, Fig. 3 (top right panel). Quantitatively, the Lagrangian characteristics turn out to be strictly related to the flow parameters, like cell size, velocity scale and eddy turnover-time, Table 2. The small discrepancies with respect to the kinematic simulations (Figs. 2, 3) are presumably due to the finite resolution of the model grid.

**Table 2:** Lagrangian characteristic quantities measured from the trajectory data analysis of the three systems: Kin2D, Delft3D and LabExp.

	$\tau _C$	$L_C$	$\lambda _L$	$D_E$
Kin2D	1 h	1 km	1.75 h$^{-1}$	0.5 km$^2$ h$^{-1}$
Delft3D	1 h	1 km	1.5 h$^{-1}$	0.4 km$^2$ h$^{-1}$
LabExp	10 s	4 cm	0.125 s$^{-1}$	1.4 cm$^2$ s$^{-1}$

See caption of Table 1 for explanation of the symbols.

### Laboratory tank experiment

The same type of 2D cellular flow was, as well, reproduced in laboratory experiments with an electromagnetically forced fluid in a non rotating tank (see Methods section for more details). By means of established experimental techniques, it was possible to reconstruct a large amount, of order $\sim 10^4$, of simultaneous Lagrangian trajectory pairs, with a time resolution of $dt_{exp}=0.2$ s, and with an initial separation equal to a fraction $\sim 0.1$ of the cell size, and long enough to last several multiples of the convective turnover time scale. A snapshot of the trajectory ensemble is plotted in Fig. 1 (lower panel).

As far as absolute dispersion is concerned, the data are consistent with the time-correlated diffusion scenario, i.e. a double scaling regime of the second-order moment, $A_2(t) \sim t^2$ in the short times limit and $A_2(t) \sim t$ in the long times limit, and auto-correlation functions shaped like damped oscillations, Fig. 2 (bottom panels). Here, slight differences with respect to the kinematic simulations are essentially due to the finite size of the tank, which limits the diffusive scale range and to the fact that the time oscillation of the fluid are controlled by a physical mechanism, which involves a smoother transition to diffusion. As regards relative dispersion, the FSLE displays a very clean picture (considering that the data come from an experiment) consisting of a small-scale plateau, below the correlation length, and a diffusive $\sim \delta ^{-2}$ scaling above the correlation length of the flow, Fig. 3 (bottom left panel). Notice the overall scale range explored by the data ($\sim$ 2 decades) is not as wide as in the model simulations, due to the limits imposed by the experimental apparatus, but still sufficient to evaluate the response of the system and the measure of physical quantities. At this regard, also in this case, the values of the Lagrangian characteristics measured from the data are consistent with the flow parameters, i.e., the spatial correlation length is of order $\sim$ 4 cm, i.e. the size of a cell around a magnet, the auto-correlation time scale is of order $\sim$ 10 s, i.e. the turnover time scale, the Lagrangian maximum Lyapunov exponent of order $\sim$ 10$^{-1}$ s$^{-1}$ and eddy-diffusion coefficient of order $\sim$ 1 cm$^2$ s$^{-1}$, Table 2.

As conclusive remark, at the end of the analysis of the three cases here considered, it is worth noting that, if the relative dispersion rates (FSLEs) are rescaled with respect to the spatial and temporal characteristic parameters of the corresponding system, Kin2D, Delft3D, and LabExp, the three curves collapse on one another, see Fig. 3, bottom right panel.

## Discussion and conclusions

We considered a regular lattice of non-stationary kinematic eddies as an example of chaotic advection and diffusion system^1–3^. This class of systems has great relevance for theoretical studies concerning, e.g., the phenomenology of Hamiltonian chaos or, in a multi-scale version, for Lagrangian turbulence modelling, as well as for applications such as sub-grid modelling of unresolved turbulent motions in Lagrangian simulations from large-scale circulation models.

The present work aimed at a new, thorough assessment of the Lagrangian dispersion properties of the chaotic advection and diffusion system, here considered, from three different perspectives: kinematic model, hydrodynamic model and laboratory experiment. Large amounts of Lagrangian trajectory pairs were analyzed, for each of these systems, in order to evaluate absolute and relative dispersion characteristics.

The laboratory tank experiment was designed to analyze one-particle and two-particle dispersion on a square lattice of magnets, covering a scale range spanning from some fraction to some multiple of the cell size, in conditions of full chaotic regime, i.e. after the destruction of any barrier or constraint to fluid particle motion due to the KAM tori. This is indeed the typical “working regime” of this kind of flow in many applications regarding Lagrangian modelling. At this regard, one of the novelties of the present study consists precisely in the experimental verification of the chaotic advection and diffusion properties for the case study here considered.

As far as the “hydro-dynamical side” of the question is concerned, another novelty consists in the Lagrangian validation test on the accuracy of the Delft3D trajectory simulations. Coastal small-scale models are usually applied to more complex flow configurations than the case study here discussed. However, unlike large-scale ocean surface model trajectories, which can be compared with real ocean drifter data, the lack of experimental or observational Lagrangian data in these complex flow configurations for coastal and/or environmental engineering applications prevents a rigorous validation of the numerical particle trajectories. On the other hand, the accuracy of Lagrangian simulations cannot be inferred only on the basis of Eulerian-type validations, since the existence of chaos implies that small differences between two velocity fields can lead to completely different trajectory evolution. The compromise was to select a simplified case study, but nevertheless displaying a non-trivial phenomenology, for which it was possible to collect a large amount of experimental data for a qualitative and quantitative assessment of the Lagrangian numerical simulations. At this regard, we would like to stress that, despite the Lagrangian validation of the Delft3D model, here presented, applies to a laminar case, the results obtained represent a first step for future testing of the model trajectory accuracy in more complex, realistic flow configurations.

## Methods

### Theoretical background

Let us define the order *n* moments of absolute and relative dispersion statistics as $A_n(t) = \langle (\mathbf{x}(t)-\mathbf{x}(0))^n \rangle$ and $R_n(t) = \langle (\mathbf{x}^{(1)}(t) - \mathbf{x}^{(2)}(t) )^n \rangle$, respectively, where $\mathbf{x}=(x_1,x_2)$ is the position of one fluid particle; $\mathbf{x}^{(1)}$ and $\mathbf{x}^{(2)}$ are the positions of two particles of a pair, *n* is a positive integer and the average $\langle \cdot \rangle$ is defined in the phase space of the system. In the present study, it will be sufficient to evaluate only the second moments, $A_2(t)$ and $R_2(t)$. Given the velocity $\mathbf{v}=(v_1, v_2)$ of a fluid particle, we consider the Lagrangian auto-correlation defined by the functions

3 $$\begin{aligned} C_{n,n}(t) = (\langle v_n(t) v_n(0) \rangle - \langle v_n \rangle ^2 )/(\langle v_n^2 \rangle - \langle v_n \rangle ^2) \end{aligned}$$

with $n=1,2$. For stationary flows, $C_{n,n}(t)$ depends only on the time lag *t*. Velocity auto-correlations relaxing to zero within a finite decay time scale $t_C$ are a necessary condition for particle dispersion to approach an asymptotic standard diffusive regime, i.e. $A_2(t) = 2 D t$ for $t \gg t_C$, where $D = \sigma ^2 t_C$ is the diffusion coefficient and $\sigma ^2$ is the velocity variance^3^.

As far as relative dispersion is concerned, the use of only time-dependent statistics is not recommendable. It is known that $R_2(t)$, for instance, when averaged over a large number of particle pairs at fixed time, can be generally affected by spurious effects that compromise the correct description of the physics of the system^22^. At this regard, the finite-scale Lyapunov exponent (FSLE), formerly introduced in the dynamical system theory for the study of non-infinitesimal perturbations^31,32^, has now become the lead scale-dependent measure of relative dispersion in various Geophysical contexts^7,8,10,11,15,22,23,33–36^. If $\delta$ is a given separation scale, $r > 1$ is a constant amplification factor and $\tau (\delta )$ is the first-exit time from shell $\delta$ to shell $r \delta$, the FSLE, $\lambda (\delta )$, is defined as:

4 $$\begin{aligned} \lambda (\delta ) = \frac{\ln (r)}{\langle \tau (\delta ) \rangle }, \end{aligned}$$

where the average $\langle \tau (\delta ) \rangle$ is measured over all particle pairs at fixed separation scale^22^. In the limit of infinitesimal separation, FSLE is expected to converge to a constant value, corresponding to the Lagrangian maximum Lyapunov exponent, i.e. $\lambda (\delta ) \rightarrow \lambda _L$; in the large-scale limit, i.e. if particle separation grows beyond the maximum correlation length of the flow, FSLE is expected to approach a standard diffusive scaling, i.e. $\lambda (\delta ) \sim \delta ^{-2}$. Henceforth, in all FSLE computations, the constant amplification ratio between consecutive scales will be set to $r=\sqrt{2}$, if not otherwise specified. The range of separation scales, $\delta _{n} = r^{n-1} \delta _{min}$, with $n=1,2,\ldots ,N_{max}$, depends on data resolution and size of the system. It has to be stressed that, although from a theoretical point of view, there is an obvious connection between the scaling properties in the temporal and spatial domains, from an application point of view, the FSLE is specifically studied to better describe the physics of dispersion that is inherently a scale-dependent process.

As a final note, we recall the basic concepts about the evolution of a passive tracer in a non linear deterministic velocity field, according to the detailed review by Crisanti et al.^3^. The full advection–diffusion equation for a passive scalar $\Theta =\Theta (\mathbf{x},t)$ in an incompressible velocity field $\mathbf{v}=\mathbf{v}(\mathbf{x},t)$ with “small-scale” diffusivity $\chi$ is:

5 $$\begin{aligned} \frac{\partial \Theta }{\partial t} + \mathbf{v} \cdot \nabla \Theta = \chi \nabla ^2 \Theta \end{aligned}$$

In our case, $\chi =0$ since we are considering a deterministic dynamics. Under certain hypotheses, depending on the form of $\mathbf{v}$, Eq. (5) can be cast into a pure diffusion equation for the average value of $\Theta$ smoothed on scale *l*:

6 $$\begin{aligned} \frac{\partial \langle \Theta \rangle _l}{\partial t} = D_E \nabla ^2 \langle \Theta \rangle _l \end{aligned}$$

where *l* is the maximum correlation length, or the maximum eddy size of the flow, in our case $l \sim L/2$ (the kinematic eddy size); $\langle \cdot \rangle _l$ represents the coarse-grain average on scale *l*; $D_E$ is defined as the effective (eddy) diffusion coefficient which accounts for the cumulative effects of the advective field on long range diffusion (even for $\chi =0$, if the Lagrangian flow is chaotic), and can be directly measured from the asymptotic separation of the trajectories:

7 $$\begin{aligned} D_E = \lim _{t \rightarrow \infty } \frac{R_2(t)}{4t} \end{aligned}$$

where the factor “4” in the denominator appears since $R_2(t)$ refers to relative dispersion. A 2D “random walk” on the lattice (due to the chaotic motion of the trajectories along the separatrices) is equivalent to a diffusion process, in the limit of spatial scales much larger than the correlation length and times much longer than the correlation time. Under these conditions, an estimate of the effective diffusion coefficient, on the basis of a dimensional argument, is given by $D_E \sim L_C^2 / \tau _C$, where $L_C$ and $\tau _C$ are of the order of the eddy size and the turnover time scale, respectively^3^.

### Delft3D

The Delft3D 4 Suite (structured) modelling software^9^ combines hydrodynamics and particle tracking processes by using two interacting modules: Delft3D-FLOW and Delft3D-PART. The computed hydrodynamic fields (Delft3D-FLOW) are used as input for the particle transport modelling (Delft3D-PART) by means of offline coupling. The Delft3D-FLOW module uses a finite difference grid and solves Reynolds Averaged Navier–Stokes equations (RANS) for an incompressible fluid in the Boussinesq approximation. It includes the horizontal momentum, continuity and transport equations, and a turbulence closure model^37^. In our specific case, we do not implement any model of turbulence and we impose zero horizontal eddy viscosity over the entire domain.

The domain consists of a square area 40 km $\times$ 40 km, with a horizontal spatial resolution equal to 100 m. The bottom is flat and is located at a constant depth of $z=-0.5$ m. The lateral walls are closed boundaries, with free slip conditions, and the initial water level is set to zero. The hydrodynamic field is forced by a time-dependent, spatially periodic wind field, formally identical to the Kin2D velocity field, with components defined by Eqs. (1) and (2). The time oscillation of the cells is driven by the wind oscillation, with oscillation amplitude equal to $\sim 10\%$ of the cell size, and oscillation period of the same order as the cell turnover time. The wind drag coefficient has constant value equal to 0.1. This means that a wind speed of $\sim$ 10 m/s generates water flow speeds of $\sim$ 1 m/s, Fig.  1 (upper and middle panels). So, the resulting hydrodynamic mean field is used as input for Delft3D-PART module, i.e. the particle tracking model. This model is based on the principle that the motion of dissolved (or particulate) substances in water can be described by a finite number of particles that are subject to flow-induced advection and, depending on the case, by horizontal and vertical diffusion (in our case this small-scale diffusion is set to zero). The advective part is solved with an analytical procedure that integrates a linearly interpolated hydrodynamic velocity field. As far as the Lagrangian simulations are concerned, the particle pairs (having the same density as the ambient water) were instantaneously released at mid-depth inside a cell located in the middle of the computational domain. The numerical integration of the particle trajectories was performed “off-line”, i.e. not simultaneous to the computation of the velocity fields. All particles were released one hour after the spin-up of the hydrodynamic computation. As a conclusive remark, we would like to stress that, since we considered particle advection due only to the mean field, i.e. filtering out the turbulent components, the Reynolds number of the flow is substantially null.

### Experimental data

**Figure 4:**
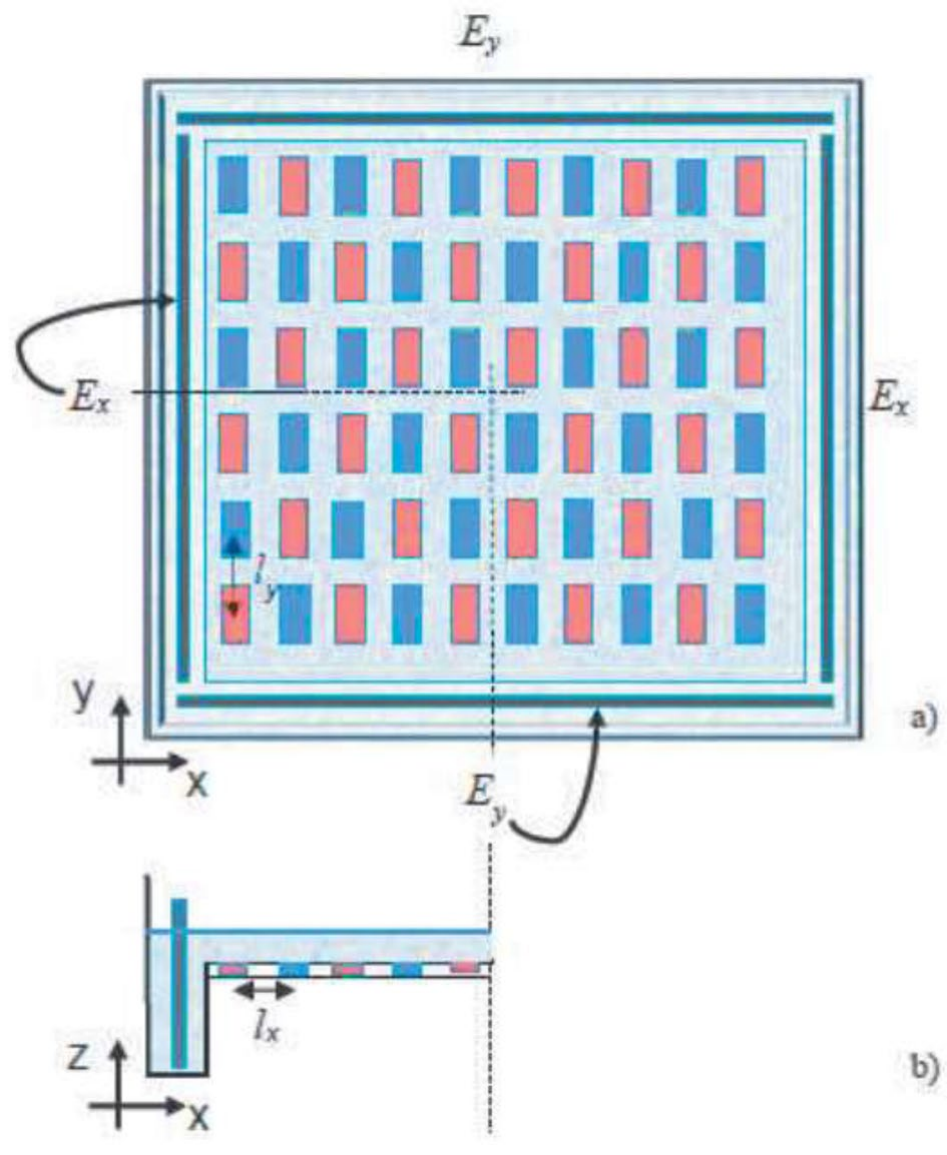
Experimental apparatus: top (**a**) and side (**b**) view; red and blue correspond to opposite (N–S) polarity; $l_x$ and $l_y$ represent the magnets’ inter-distance along the x and y direction; currents *I* and $I^{'}$ are generated by the electrodes $E_x$ and $E_y$, respectively.

The experimental set-up, Fig. 4, consists of a square plexiglas tank of side $L_0 = 50$ cm and height $H_0 = 5$ cm, partially filled with a thin layer of an electrolyte solution of water and NaCl (density $\rho \sim$ 1060 g/l, concentration of NaCl = 50 g/l). The flow is generated by means of electromagnetic forcing, that is, via the Lorentz force arising from the interaction of electric and magnetic fields^38^.

This type of fluid forcing represents a valid tool to easily simulate and control 2D (or almost 2D) flows since it allows to study different configurations and patterns by varying salinity, intensity of the current, and magnets’ positioning; similar arrangements have widely used to study diffusion and mixing properties in 2D flows^19,20,39^ and 2D turbulence^40^. The system’s rotation can be simulated making the setup particularly suitable to study atmospheric and oceanic flows^41–44^.

In general, intensity and stability of the vortices depend on the applied forcing; in continuously forced conditions, the subsequent nonlinear interaction of the flow structures may result in quasi-2D turbulence. In our set of experiments, we considered two orthogonal electric currents *I* and $I'$, driven in the horizontal $x{-}y$ (measurements) plane and a magnetic field along the z-(vertical) direction. In particular, currents were generated by connecting two couples of titanium electrodes ($E_x, E_y$) to a power supply (Fig. 4): along the *x*-axis the current *I* is constant in time while, in the orthogonal direction, $I'$ varies according to a sinusoidal law of frequency *f*. The values considered in these experiments varied, respectively, in the ranges: 2 A $\le I-I' \le$ 4 A, 0.04 Hz $\le f \le 0.1$ Hz.

To generate the magnetic field we placed an array of rectangular permanent (neodymium) magnets on a metallic plate located 5mm underneath the tank bottom. Dimensions of the magnets and the strength of the magnetic field, measured above the magnets surface, are $L_x \times L_y \times H_m = 20 \times 10 \times 5\,\mathrm{mm}^3$ and $B \sim 1232$ G, respectively. They were arranged with alternating polarity along x and y axis in the horizontal plane and spaced of 5 mm along both directions (i.e. the centre-centre distance is $l_x =15$ mm, $l_y=25$ mm). With this configuration, when the current $I^{'}$ is null, the flow pattern is characterized by opposite-signed vortices, clockwise or anti-clockwise according to the phase of the resulting Lorentz force, whose horizontal length scale is related to the magnets’ inter-distance and to their size. When $I^{'}$ is switched on, the resulting flow pattern is consistent with the time oscillating stream-function defined in (2).

**Figure 5:**
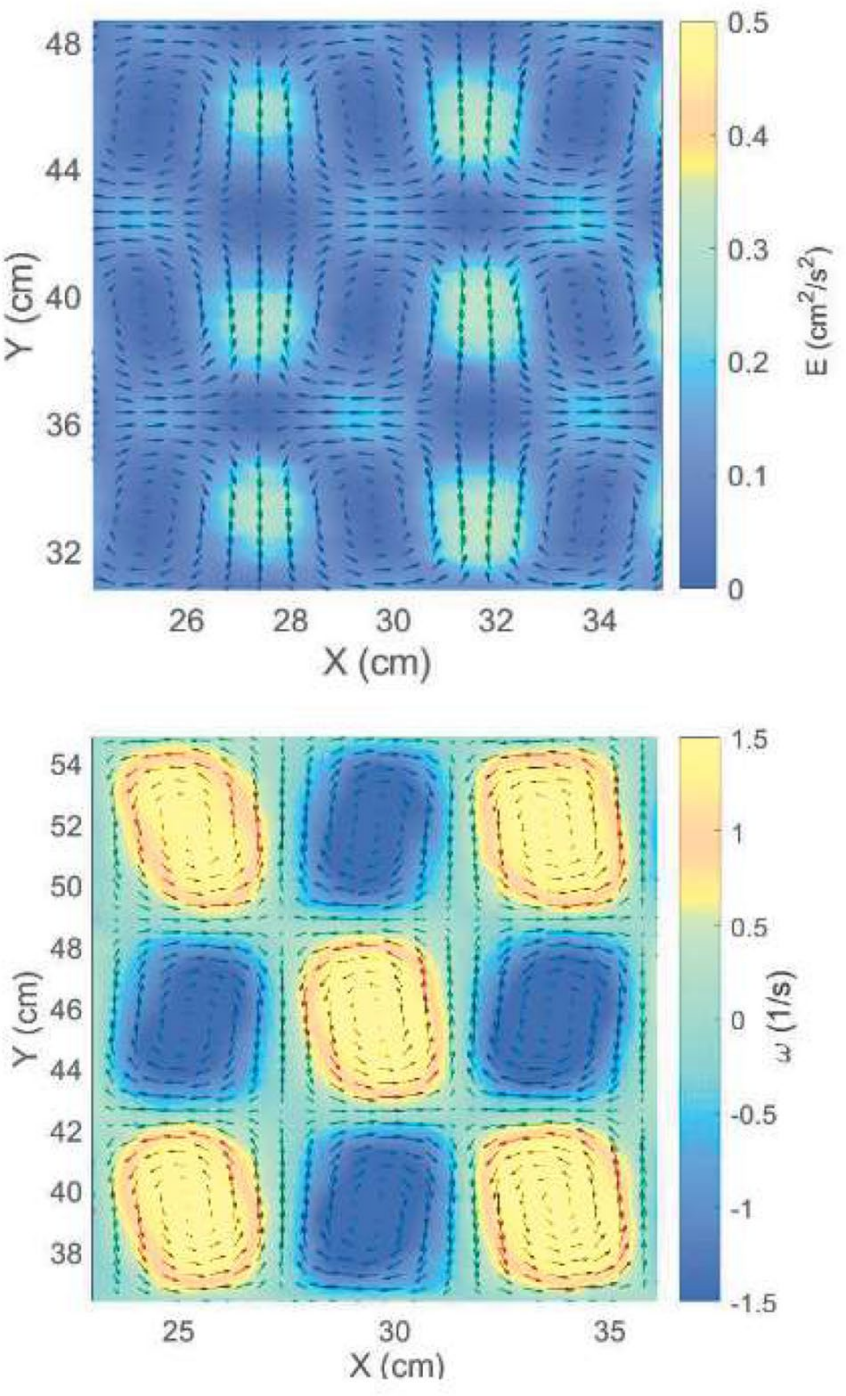
Time averaged velocity field overlapped to kinetic energy (top) and vorticity (bottom) maps for the set up: $I = 2$ A, $I^{'} = 1$ A, $f = 0.08$ Hz. Velocity data are interpolated on a $128\times 128$ grid.

The fluid flow is measured by using a Feature Tracking (FT) image analysis, a technique that allows for a description of the fluid motion in a Lagrangian framework^45^. To this aim, the fluid surface is seeded with buoyant styrene particles (density $\sim$ 1 g/cm$^3$, mean diameter $d_p =$ 50 $\upmu$m; tracers are supposed to be passively advected by the flow) and lit with two lateral lamps to gain a high contrast between the white particles and the black bottom. After the forcing is activated, flow images are acquired by a video camera perpendicular to the tank (dimension of the framed area: $1000 \times 1000$, acquisition rate $20{-}25$ fps). Image processing is achieved in three subsequent steps: (i) pre-processing aimed at removing the background and improving image contrast; (ii) particle detection and tracking to obtain the flow description; (iii) post-processing to obtain the relevant flow parameters. The sparse velocity vectors (i.e. along each trajectory) are detected in each frame of the acquired time sequence. Subsequently, they can be interpolated onto a regular grid and the time evolution of the Eulerian flow field and all the derived quantities (i.e. vorticity, kinetic energy, etc.) can be obtained as well, Fig. 5. Unlike numerical models, here the turbulent components of the flow cannot be filtered out from the mean field. An estimate of the Reynolds number can be obtained by multiplying typical size, $\sim$ O(1) cm, and rotation velocity, $\sim$ O(1) cm/s, of the eddies, divided by the kinematic viscosity of the fluid, $\sim$ 10$^{-6}$ m$^2$/s: Re $\sim$ O(10$^2$), far below the critical threshold for the onset of fully developed turbulence.

## Data Availability

The datasets used and/or analysed during this study are available from the corresponding author on reasonable request.
